# Development of an industrial yeast strain for efficient production of 2,3-butanediol

**DOI:** 10.1186/s12934-022-01924-z

**Published:** 2022-09-29

**Authors:** Guangxin Huo, María R. Foulquié-Moreno, Johan M. Thevelein

**Affiliations:** 1 Laboratory of Molecular Cell Biology, Institute of Botany and Microbiology, KU Leuven, Leuven-Heverlee, Belgium; 2 Center for Microbiology, VIB, Kasteelpark Arenberg 31, B-3001 Leuven-Heverlee, Flanders Belgium; 3 NovelYeast Bv, Open Bio-Incubator, Erasmus High School, Laarbeeklaan 121, B-1090 Brussels (Jette), Belgium

**Keywords:** Yeast cell factory, Bio-based chemical, Metabolic engineering, 2,3-Butanediol, Alternative oxidase, NADH oxidase

## Abstract

As part of the transition from a fossil resources-based economy to a bio-based economy, the production of platform chemicals by microbial cell factories has gained strong interest. 2,3-butanediol (2,3-BDO) has various industrial applications, but its production by microbial fermentation poses multiple challenges. We have engineered the bacterial 2,3-BDO synthesis pathway, composed of *AlsS*, *AlsD* and *BdhA*, in a pdc-negative version of an industrial *Saccharomyces cerevisiae* yeast strain. The high concentration of glycerol caused by the excess NADH produced in the pathway from glucose to 2,3-BDO was eliminated by overexpression of NoxE and also in a novel way by combined overexpression of *NDE1*, encoding mitochondrial external NADH dehydrogenase, and *AOX1*, encoding a heterologous alternative oxidase expressed inside the mitochondria. This was combined with strong downregulation of *GPD1* and deletion of *GPD2*, to minimize glycerol production while maintaining osmotolerance. The HGS50 strain produced a 2,3-BDO titer of 121.04 g/L from 250 g/L glucose, the highest ever reported in batch fermentation, with a productivity of 1.57 g/L.h (0.08 g/L.h per gCDW) and a yield of 0.48 g/g glucose or with 96% the closest to the maximum theoretical yield ever reported. Expression of *Lactococcus lactis* NoxE, encoding a water-forming NADH oxidase, combined with similar genetic modifications, as well as expression of *Candida albicans STL1*, also minimized glycerol production while maintaining high osmotolerance. The HGS37 strain produced 130.64 g/L 2,3-BDO from 280 g/L glucose, with productivity of 1.58 g/L.h (0.11 g/L.h per gCDW). Both strains reach combined performance criteria adequate for industrial implementation.

## Introduction

2,3-butanediol (2,3-BDO) is a platform chemical of which several structural derivatives have multiple industrial applications [1, 2]. 2,3-BDO has 2 chiral carbon atoms and exists as liquid without color and odor at room temperature [3]. Due to the structure of 2,3-BDO and its two hydroxyls, several chemical reactions, including dehydration, dehydrogenation, ketalization and esterification, provide a range of possibilities to produce many 2,3-BDO derivatives [4]. 1,3-butadiene can be produced from 2,3-BDO through dehydration creating double bonds in a one-step reaction. It is used as the monomer for production of synthetic rubber [5]. 2,3-BDO can also be converted into an attractive fuel additive, methyl ethyl ketone (MEK), via pinacol rearrangement under acidic conditions [6]. 2,3-BDO can be used as an important component of antifreeze agents because of its low freezing point (− 60 °C) [7]. Currently, commercial 2,3-BDO is produced by chemical processes from petroleum through hydrolysis of 2,3-butane oxide in an environmentally-unfriendly energy intensive process [8, 9]. Expensive catalysts and the high energy demand result in a high price for 2,3-BDO ($1600/t), which limits availability and restricts the global market [10].

Nowadays, global warming caused by the huge emissions of CO_2_, the widespread environmental pollution as well as the increasing petroleum prices have stimulated interest in producing bio-based chemicals from biomass [11–13]. Microbial 2,3-BDO production is a more sustainable process that is friendly to the environment and reduces both CO_2_ emissions and energy costs. Therefore, it has gained increasing attention in recent years [8, 14, 15]. Various microorganisms, including bacteria and yeast, have been used for 2,3-BDO production [1, 2, 16]. A high 2,3-BDO titer, about 150 g/L, with a high productivity (3.95 g L^−1^ h^−1^), has been achieved by using a wild type *Klebsiella pneumoniae* strain with an optimized fermentation medium and conditions [17]. *Bacillus. licheniformis* DSM 8785 is also an interesting 2,3-BDO producer supporting a high titer of 144.7 g/L in a fed batch fermentation [18]. 2,3-BDO is an important secondary metabolite produced for preventing acidification and for carbon source storage by *Klebsiella pneumoniae* KCTC2242. Optimized expression of the genes involved in the 2,3-BDO biosynthetic pathway in *K. pneumoniae* increased the 2,3-BDO titer to 1.6 fold of that in the parental strain. It reached 101.53 g/L after 40 h fermentation with a productivity of 2.54 g L^−1^ h^−1^ in the strain with overexpression of *budA* (acetolactate decarboxylase) and *budB* (acetolactate synthase) [19]. Recently, a mutant *Enterobacter ludwigii* strain has been developed for 2,3-BDO production by using Brewers’ spent grain hydrolysate as feedstock, which finally resulted in a titer of 118.5 g/L 2,3-BDO and a high yield of 0.48 g/g glucose [20].

Most of the bacteria produced 2,3-BDO from different carbon sources with a mixed acid fermentation, which generates several different products, such as acetic acid, ethanol and lactic acid [4, 21, 22]. Blocking the metabolic pathways producing the by-products redirects more carbon source to 2,3-BDO production and constitutes the most efficient, but lengthy way to reach a high 2,3-BDO yield, which is a major requirement for industrial application [23]. The *ldhA* gene, encoding lactate dehydrogenase, was identified by flux balance analysis (FBA) in *K. oxytoca* as the best candidate gene for single deletion to reduce lactate production and increase the 2,3-BDO yield [24]. Acetoin, the precursor of 2,3-BDO, also constitutes a by-product, but only when there is a shortage of sufficient NADH [25]. The elimination of NADH oxidase (*YodC*) and heterologous expression of a formate dehydrogenase gene in *Bacillus subtilis* provided more NADH for 2,3-BDO production and led to less acetoin accumulation [26]. In addition, deletion of the *pta* and *ldh* genes eliminated acetate and lactate production, respectively. This was followed by deletion of the D-(–)− 2,3-BDO dehydrogenase gene (*bdhA*), and expression of the meso-2,3-BDO dehydrogenase gene (*budC*), from *K. pneumoniae*. This engineered *B. subtilis* strain produced 103.7 g/L pure meso-2,3-BDO in a semi-aerobic condition with a yield of 0.487 g/g [27].

The production of 2,3-BDO with bacteria is often cumbersome, because of pathogenicity, low robustness, phage sensitivity and difficulty to combat contamination with other bacteria [23]. *Saccharomyces cerevisiae* is a promising alternative host for industrial production of 2,3-BDO since it does not suffer from these shortcomings [1, 2] and has been used extensively for large scale commercial production of bioethanol as well as many other bio-based chemicals [28–30]. As pyruvate is a precursor for both alcoholic fermentation and 2,3-BDO biosynthesis, it is crucial to block ethanol production in order to shift the carbon flux to 2,3-BDO synthesis [31]. This can be done by deletion of alcohol dehydrogenase or pyruvate decarboxylase genes in a strain with expression of the bacterial 2,3-BDO synthesis pathway [32, 33]. A pyruvate decarboxylase (Pdc) deleted *S. cerevisiae* strain was engineered with introduction of acetolactate synthase (*alsS*) and acetolactate decarboxylase (*alsD*) from *B. subtilis* and overexpression of the endogenous 2,3-BDO dehydrogenase 1 (encoded by *BDH1*). The resulting strain produced 96.2 g/L 2,3-BDO in 244 h with a yield of 0.28 g/g glucose under oxygen-limiting conditions [32]. Deletion of the *PDC* genes, however, creates a C2-auxotrophy because of deficiency in cytosolic acetaldehyde production. This can be overcome to some extent by provision of a C2-compound like ethanol in the medium or by evolutionary adaptation [34].

Glycerol is the main by-product of 2,3-BDO production in *S. cerevisiae* strains in which ethanol production has been eliminated and this is due to the excess NADH generated during the conversion process from glucose to 2,3-BDO [35]. In 2,3-BDO production, 1 mol glucose is converted into 2 mol pyruvate and 2 mol NADH via glycolysis, and 2 mol pyruvate is then used to synthesize 1 mol 2,3-BDO with only 1 mol NADH being re-oxidized, which leads to an excess of 1 mol NADH per mole of glucose consumed [21]. As a result, a large amount of glycerol is produced as a sink for the surplus NADH to keep the redox balance [36]. This problem has been addressed up to now only by expressing the bacterial *NoxE* encoded water-forming NADH oxidase. With NoxE from *Lactococcus lactis* the 2,3-BDO yield increased to 0.359 g/g glucose and decreased the glycerol yield to 0.069 g/g glucose since part of the excess NADH was consumed by oxygen instead of generating glycerol [37]. The engineered *S. cerevisiae* strain with elimination of alcohol dehydrogenase (*adh1-5*) and NAD-dependent glycerol 3-phosphate dehydrogenase (*gpd1,2*), and expressing the *NoxE* gene to oxidize excess NADH and maintain the redox balance, resulted in a yield for 2,3-BDO production of 0.41 g/g glucose and no glycerol synthesis [35]. *Candida tropicalis PDC1* was used to produce ethanol as essential cytosolic C_2_ compound to maximize cell growth and 2,3-BDO production in the pdc-deficient *S. cerevisiae* strain with expression of the *NoxE* gene to lower glycerol production. The engineered strain produced 154.3 g/L 2,3-BDO in 78 h with a yield of 0.404 g/g glucose [38].

Alternative oxidase is a cyanide-insensitive terminal oxidase integrated in the inner mitochondrial membrane in all plants, most fungi, algae and some protists. It catalyzes the four-electron transfer from ubiquinol (reduced form of ubiquinone) to oxygen to generate water with release of only heat and no ATP generation [39]. The expression of the alternative oxidase from other species in *S. cerevisiae* mitochondria was shown to reduce the overflow of NADH under aerobic conditions of high glycolytic flux [40, 41]. The alternative oxidase has not been used yet to overcome the excess redox power in 2,3- BDO production, possibly because the huge excess of NADH is generated in the cytosol, which creates a challenge for its oxidation by alternative oxidase in the mitochondria.

In this study, we have created a new NADH oxidation pathway by overexpression of the homologous mitochondrial external NADH dehydrogenase (Nde1, Saccharomyces Genome Database ID: S000004753; EC Number 1.6.5.9) [42] and the alternative oxidase (Aox1, UniProtKB –Q9Y711; EC Number 1.10.3.11) from *Histoplasma capsulatum* in *S. cerevisiae* [43]. Through this pathway, Nde1 enhances the transfer of electrons from cytosolic NADH into the ubiquinol pool which is then oxidized with oxygen by the alternative oxidase. Both this new alternative oxidase pathway and the *NoxE* pathway have been explored in parallel in this study to oxidize excess NADH of the 2,3-BDO production in order to reduce glycerol formation. Both strategies were shown to be effective in NADH re-oxidation. Together with several other genetic modifications, the two strategies resulted in two top performance yeast strains displaying a combination of high yield, high titer and high productivity for 2,3-BDO production suitable for industrial implementation.

## Methods

### Strains and plasmids

The *S. cerevisiae* strains used in this work have been listed in Table 1, while the plasmids used have been listed in Table 2.

**Table 1:** *S. cerevisiae* strains used in this work

Strain	Genotype	Reference
GSE16-T18HAA1	Industrial 2G xylose-fermenting and inhibitor-tolerant strain	MCB, KULeuven [44]
HGS1	GSE16-T18 *pdc1::* BDOp BDOp:2,3-BDO pathway being TDH3p-AlsD-ADH1t, ADH1p-AlsS-ADH2t, TEF1p-BdhA-CYC1t	This study
HGS2	HGS1 pdc6*::* BDOp BDOp:2,3-BDO pathway being TDH3p-AlsD-ADH1t, ADH1p-AlsS-ADH2t, TEF1p-BdhA-CYC1t	This study
HGS4	HGS2 pdc5*::* BDOp BDOp:2,3-BDO pathway being TDH3p-AlsD-ADH1t, ADH1p-AlsS-ADH2t, TEF1p-BdhA-CYC1t	This study
HGS7	HGS4 AD7:: TDH3p-BdhA-ADH1t, ADH1p-BdhA-ADH2t AD7, integration site	This study
HGS21	HGS7 mk114:: TDH3p-Aox1-ADH1t, ADH1p-Nde1-ADH2t, Mk114, integration site	This study
HGS31	HGS21 GPD2:: GPD2p-Nde1-GPD2t Nde1 replaces GPD2	This study
HGS38	HGS31 GPD1:: GPD1p-Nde1-GPD1t Nde1 replaces GPD1	This study
HGS43	HGS38 *Mpc1 ∆* Mpc1 deletion	This study
HGS48	HGS43 *Ora1∆* *Ora1* deletion	This study
HGS50	HGS48 Mk20:: CYC1p-GPD1-GPD1t Mk20, integration site	This study
HGS8	HGS7 mk114:: TDH3p-Aox1-ADH1t Mk114, integration site	This study
HGS15	HGS8 *GPD1∆*, *GPD2∆* *GPD1* and *GPD2* deletion	This study
HGS17	HGS8 GPD1p::CYC1p, *GPD2∆* GPD1p replaced by CYC1p,*GPD2* deletion	
HGS28	HGS15 mk20:: CYC1p-GPD1-GPD1t Mk20,integration site	This study
HGS29	HGS28 *Ora1 ∆* *Ora1* deletion	This study
HGS37	HGS29 Mk119:: TEF1p-CSTL1-CYC1t Mk119, integration site	This study

*CSTL1* Gene encoding sugar transporter-like (Stl1) protein from *Candida albicans*

**Table 2:** Plasmids used in this work

p426-hph	pMB1 ori (*E. coli*) and 2 micron ori (S. cerevisiae, multi-copy), hph marker backbone for construction of donor DNA	MCB, KU Leuven
pBEVY-hph	ColE1 ori (*E. coli*) and 2 micron ori (*S. cerevisiae*, multi-copy), hph marker backbone for construction of BDOp	MCB, KU Leuven
pTEF-Cas9-KanMX (p51)	pBR322 ori (*E. coli*) and CEN ori (single copy), vector backbone p414-TEF1p-Cas9-CYC1t KanMX marker	MCB, KU Leuven
pgRNA-uni-hph (p58)	pBR322 ori (*E. coli*) and 2 micron ori (*S. cerevisiae*, multi-copy) gRNA plasmid backbone with hph marker	MCB, KU Leuven
pgRNA-uni-NAT (p59)	pBR322 ori (*E. coli*) and 2 micron ori (*S. cerevisiae*, multi-copy) gRNA plasmid backbone with NAT marker	MCB, KU Leuven
P58-PDC1	P58 backbone with 2 gRNA targeting sequence for PDC1 (AGCATCCAACAATTTTTGCA and GATAAGCTTTATGAAGTCAA)	MCB, KU Leuven
P58-PDC5	P58 backbone with 2 gRNA targeting sequence for PDC5 (AGCATCCAACAATTTTTGCA and GATAAGCTTTATGAAGTCAA)	MCB, KU Leuven
P58-PDC6	P58 backbone with 2 gRNA targeting sequence for PDC6 (CTATCGAAAAGCTGATTCAT and GCTGATTTGATCCTTTCGGT	This study
P59-mk114	P59 backbone with 2 gRNA targeting sequence for mk114 site (GTGATTTCGTGTGCAACCAA and TGACAACAAAGAAGCAAATA)	This study
P59-GPD2	P59 backbone with 2 gRNA targeting sequence for GPD2 (CACCATCGCCAAAGTCATTG and CTCCGCAGCCATTCAAAGGC)	This study
P59-GPD1	P59 backbone with 2 gRNA targeting sequence for GPD1 (CGTATCTGTAGCCAATTGAA and AGTGTCATCGAAGATGTTGC)	This study
P59-MPC1	P59 backbone with 2 gRNA targeting sequence for MPC1 gene (AAAGACCCTACACTAATCTC and GAAACTGCGCAATTAGCTCA)	This study
P59-Ora1	P59 backbone with 2 gRNA targeting sequence for Ora1 gene (AAGAAGACTGTCCTCATTAC and AGTTAGATACAGAGGTAACG)	This study
P59-mk20	P59 backbone with 2 gRNA targeting sequence for mk20 site (TCGAATCCAGAATCAGATAC and GCCGTTCAGTCGAAAGAGTT)	This study
pBEVY-BDOp-PDC1	pBEVY backbone with BDOp and homologous regions for integration at PDC1 locus	This study
pBEVY-BDOp-PDC5	pBEVY backbone with BDOp and homologous regions for integration at PDC5 locus	This study
pBEVY-BDOp-PDC6	pBEVY backbone with BDOp and homologous regions for integration at PDC6 locus	This study
pBEVY-bdhA-AD7	pBEVY backbone with expression cassette TEF1p-BdhA-CYC1t and homologous regions for integration at AD7 site	This study
pBEVY-Nde1-Aox1-mk114	pBEVY backbone with TDH3p-Nde1-ADH1t, ADH1p-Aox1-ADH2t and homologous regions for integration at mk114 site	This study
pBEVY-Nde1-GPD2	pBEVY backbone with Nde and homologous regions for replacement of GPD2 gene	This study
pBEVY-Nde1-GPD1	pBEVY backbone with Nde and homologous regions for replacement of GPD1 gene	This study
pBEVY-CYC1p-GPD1-mk20	pBEVY backbone with CYC1p-GPD1-GPD1t and homologous regions for integration of mk20 site	This study
P426-NoxE-mk114	P426 backbone with TDH3p-NoxE-ADH1t and homologous regions for integration of mk114 site	This study
pBEVY-CSTL1-mk119	pBEVY backbone with TEF1p-CSTL1-CYC1t and homologous regions for integration of mk119 site	This study

### Genomic DNA and plasmid extraction

Genomic DNA of yeast cells for PCR or sequence analysis was extracted by the phenol/chloroform/isoamylalcohol (25:24:1) method. The precipitated DNA was washed twice with ethanol and finally dissolved in MilliQ water. Plasmids were extracted with the commercial Nucleospin Plasmid EasyPure kit (Macherey Nagel) according to the protocol provided by the manufacturer. The plasmids were digested with relevant restriction enzymes to ligate DNA fragments via Gibson Assembly followed by transformation in *E. coli* Top10 cells and the transformants were selected on LB agar plates with ampicillin. The transformants were checked by colony PCR with Standard Taq polymerase to select positive colonies. Two positive colonies were inoculated into LB medium with ampicillin and grown overnight for plasmid extraction. Plasmids were sent for sequence analysis after purification according to the protocol provided by the manufacturer.

### Media and growth conditions

Yeast cells were grown in YPD medium (10 g/L yeast extract, 20 g/L bacteriological peptone and 20 g/L glucose) at 30 °C in a rotary shaker at 200 rpm. For culturing of *pdc*-negative strains, 5 g/L ethanol was supplied. Solid YPD plates were obtained by adding 15 g/L Bacto™ agar in the media. Transformants were selected on solid YPD plates with one antibiotic or a combination of different antibiotics (200 mg/L geneticin, 300 mg/L hygromycin or 200 mg/L nourseothricin) in a 30 °C incubator. For long-term storage of engineered strains at − 80 °C, 300 μL glycerol (87%) was mixed with 700 μL culture medium. Plasmid construction and multiplication in *E. coli* (Top10) was carried out in lysogeny broth (LB) medium supplemented with 100 mg/L ampicillin and incubated at 37 °C. To obtain solid LB nutrient plates, 15 g/L Bacto™ agar was added to LB medium.

### Strain construction

The lithium-acetate method or electroporation was used for yeast transformation. Gene deletion or integration was carried using the CRISPR/Cas9 genome editing system. Yeast cells were first transformed with Cas9 enzyme expression plasmid (p51) and selected on YPD(E) solid plates with geneticin. The yeast cells harboring p51 were then precultured in YPD(E) medium with geneticin for the second transformation with the gRNA plasmid and the linear donor. The transformants were selected on YPD(E) solid nutrient plates with relevant antibiotics for the p51 and gRNA plasmids. Three positive colonies were inoculated in YPD(E) medium after confirmation with PCR using Standard Taq polymerase and transferred three times in YPD(E) medium to lose the plasmids.

### Gene synthesis and PCR

The Open Reading Frame (ORF) of the following genes was codon optimized and sent for synthesis at IDT: α-acetolactate synthase (*AlsS*, Gene ID: 936,852), α-acetolactate decarboxylase (*AlsD,* Gene ID: 936,857), butanediol dehydrogenase (*bdhA*, Gene ID: 939,490) and alternative oxidase (*Aox1*, UniProtKB -Q9Y711). The homologous NAD-dependent glycerol-3-phosphate dehydrogenase (*GPD1*) and Sugar Transporter-Like protein (*STL1*) from *Candida albicans* were directly amplified from respective genomic DNA. The above genes were amplified with high-fidelity polymerase Q5 (New England Biolabs) and the amplicons were purified with Wizard SV Gel and PCR clean-up kit (Promega) and prepared for cloning into vectors using the Gibson assembly master mix (New England Biolabs). The donor DNA for gene deletion was constructed via two PCR amplifications. The upstream and downstream sequence (about 400 bp) of the ORF were amplified with high-fidelity polymerase Q5, with an overlap of 40 bp and purified for the second round of PCR (fusion PCR), which was then purified for use as donor DNA.

### 2,3-BDO fermentation and analysis

Yeast cells were inoculated in glass tubes with 3 mL YPD(E) medium from plates and grown to stationary phase. Then 2 mL of cell culture was transferred into a 300 mL erlenmeyer with 50 mL 10% YPD(E) medium and cultured for 16-20 h. A certain volume of cells was centrifuged and resuspended with fermentation medium and then inoculated in a 300 mL fermentation tube or 300 mL erlenmeyer containing 50 mL fermentation medium with starting OD_600_ = 5. The strains HGS4 and HGS7 were cultured in fermentation tubes under semi-anaerobic conditions because they use glycerol as the final electron acceptor while the fermentation of the other strains was done in erlenmeyers under aerobic conditions because they use oxygen as final electron acceptor. All strains were grown in triplicate and samples for analysis were taken at different time points. The supernatant of the samples was diluted so that the final concentration of the sugar and metabolites ranged from 0 g/L to 10 g/L. 10 μL of the diluted sample was injected in the mobile phase (5 mM H_2_SO_4_) with a flow rate of 0.7 mL/min. The compounds were separated on a Biorad Aminex HPX 300 × 7.8 mm column, maintained at 60 °C, and detected with a UV detector (SPD-20A, Shimadzu) and RID detector (RID-20A, Shimadzu).

## Results

### Elimination of ethanol production

**Fig. 1:**
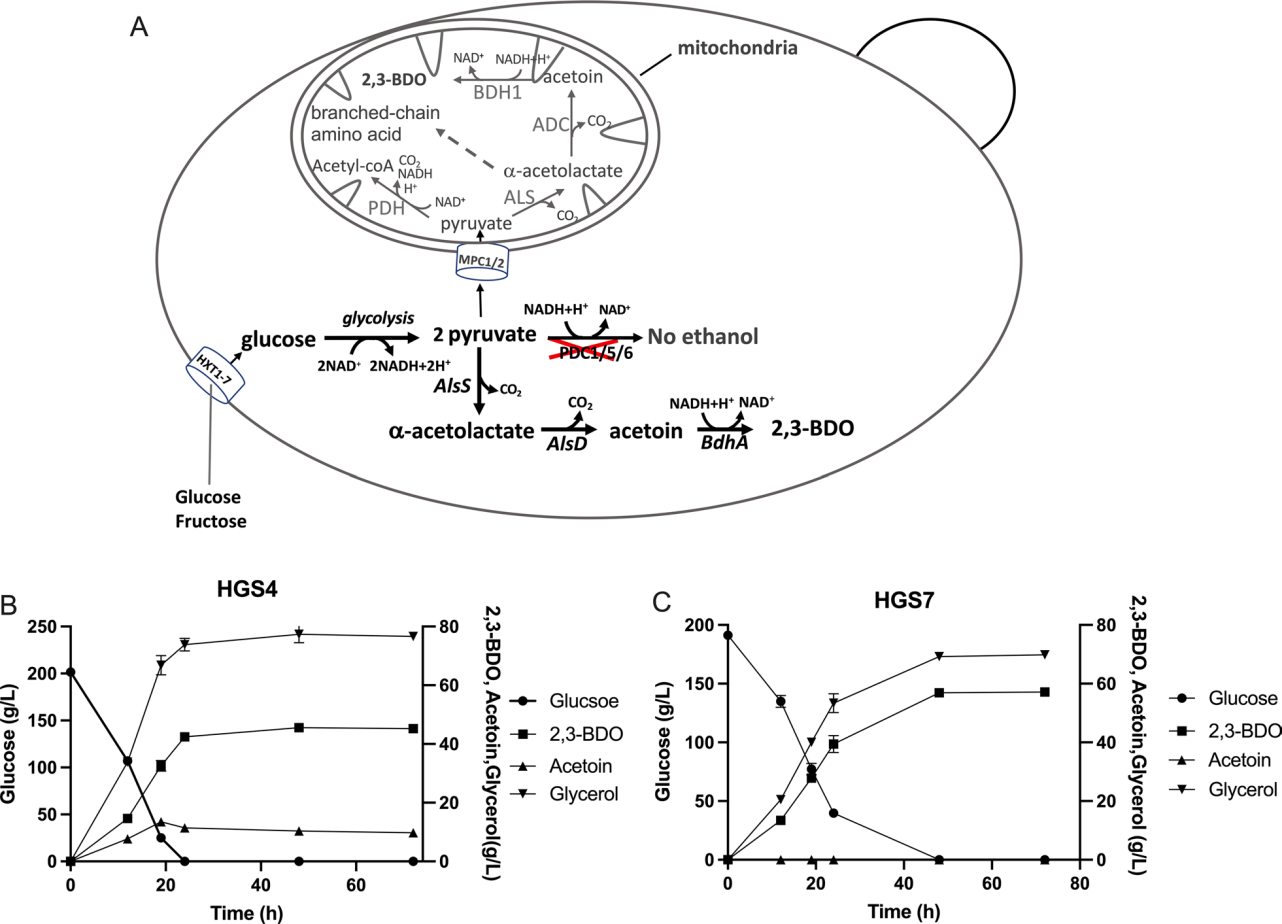
Endogenous and engineered 2,3-BDO production in yeast. **A** Endogenous mitochondrial 2,3-BDO synthesis pathway and engineered heterologous cytosolic 2,3-BDO production pathway in *S. cerevisiae*. **B** Production of 2,3-BDO from glucose by the *PDC*-negative strain HGS4 engineered with the 2,3-BDO synthesis pathway. **C** Production of 2,3-BDO from glucose by the *PDC*-negative strain HGS7 overexpressing *bdhA*. The fermentation was performed in 50 ml medium in a static fermentation tube

An overview of the native and engineered metabolic pathway for 2,3-butanediol production is shown in Fig. 1A.

The *PDC1*, *PDC5* and *PDC6* genes were deleted in strain HGS4 and each gene was replaced with a construct encoding the three 2,3-BDO pathway genes: *AlsS*, encoding α-acetolactate synthase, *AlsD*, encoding α-acetolactate decarboxylase, and *BdhA*, encoding acetoin reductase. The fermentation profile of the resulting HGS4 strain is shown in Fig. 1B. Glucose (200 g/L) was consumed in 24 h and 45.26 g/L 2,3-BDO produced in the same period. On the other hand, the strain also produced 76.63 g/L glycerol and 9.75 g/L acetoin at the same time.

### Downregulation of acetoin production

**Fig. 2:**
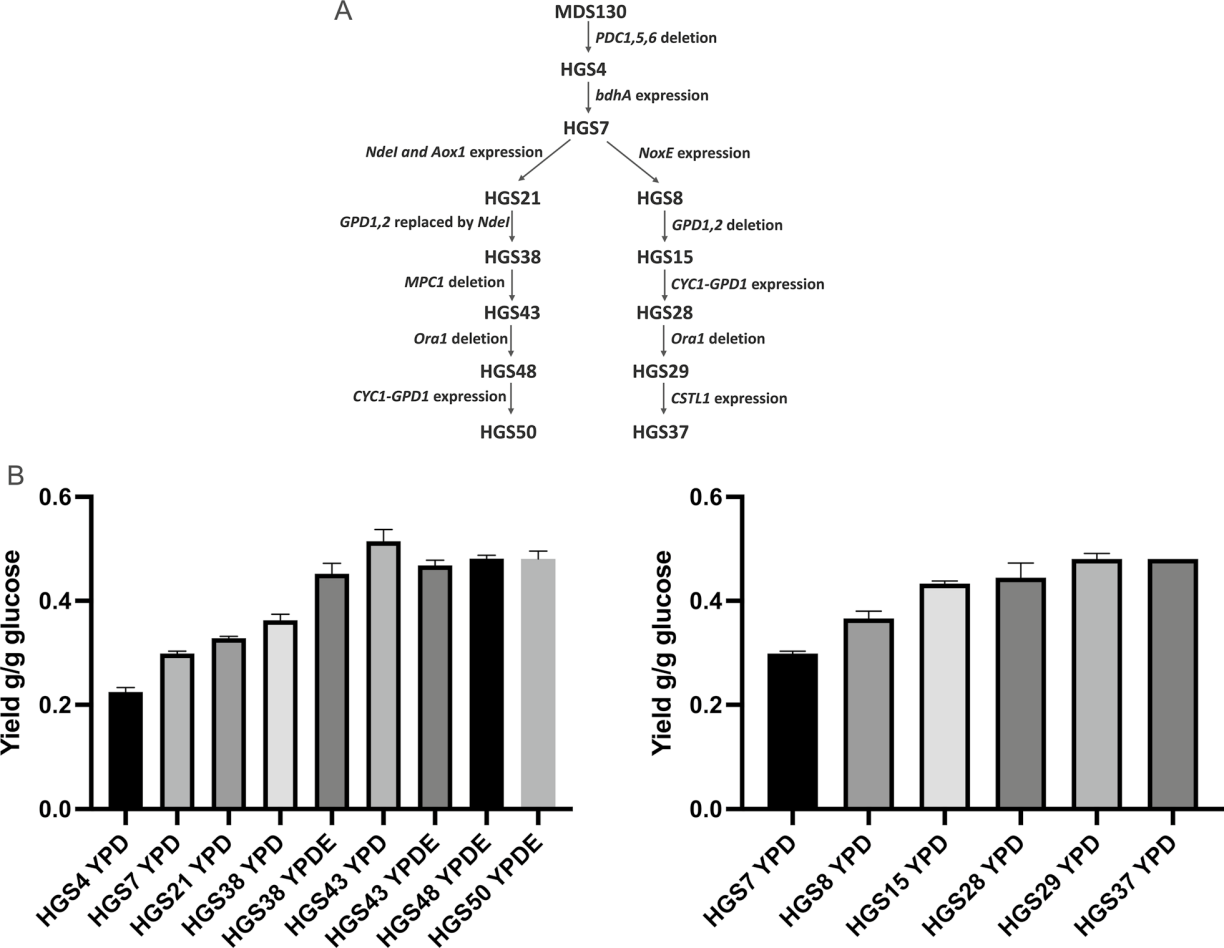
Strain lineage and increase in 2,3-BDO production yield in the two parallel series of strains. **A** Lineage of strain construction in the two parallel series. **B** The yield increased in the first series from HGS4 till HGS50 and in the second series from HGS7 till HGS37

To stimulate the conversion of the intermediate acetoin, we next overexpressed the *BdhA* gene by insertion of two copies in a single construct, using the *TDH3* promoter and *ADH1* promoter, into the AD7 integration site [45]. The resulting strain HGS7 produced a higher 2,3-BDO level (57.17 g/L) and no significant amount of acetoin, but glucose consumption was somewhat slowed down and glycerol production remained highly elevated at 69.80 g/L (Fig. 1C). The yield of 2,3-BDO production increased from about 0.23 to about 0.30 g 2,3-BDO/g glucose (Fig. 2). This fermentation was performed in a static fermentation tube.

### Downregulation of glycerol production using an alternative oxidase mitochondrial pathway for NADH consumption

**Fig. 3:**
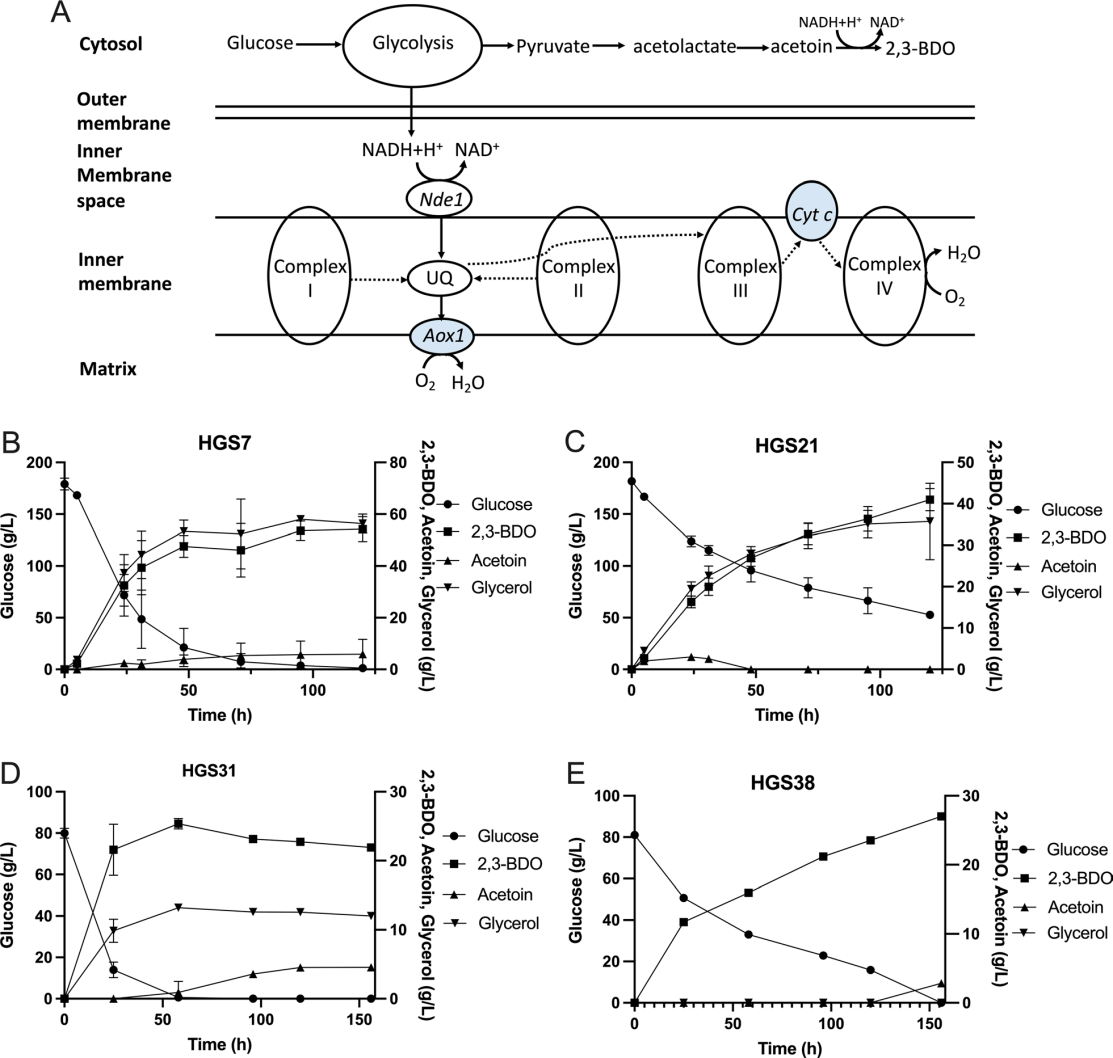
Reduction of glycerol production by expression of an alternative oxidase pathway. **A** Scheme of the alternative oxidase pathway comprising the mitochondrial external NADH oxidase Nde1 and the heterologous inner membrane enzyme Aox1, which shuttles reducing equivalents directly from ubiquinone (UQ) to oxygen. **B** Production of 2,3-BDO from glucose by the *PDC*-negative strain HGS7 overexpressing *bdhA*. The fermentation was performed in 50 ml medium in a shake flask. **C** Production of 2,3-BDO from glucose by strain HGS21 expressing Nde1 and Aox1. The fermentation was performed in 50 ml medium in a shake flask. **D** Production of 2,3-BDO from glucose by strain HGS31 in which *GPD2* is replaced by *NDE1*. **E** Production of 2,3-BDO from glucose by strain HGS38 in which both *GPD1* and *GPD2* are replaced by *NDE1*

To improve the performance of the HGS7 strain, two parallel approaches were evaluated to address the excess of NADH generated (Fig. 2A). First we have tried a novel approach in which the *NDE1* gene, encoding mitochondrial external NADH dehydrogenase, and the *AOX1* gene, encoding a heterologous alternative oxidase from *Histoplasma capsulatum*, were expressed using the *ADH1* and *TDH3* promoter, respectively, in the Mk114 integration site [46]. Expression of both genes together creates an alternative pathway for NADH consumption (see scheme in Fig. 3A), which aims to reduce glycerol production. To support sufficient oxygen provision for the alternative NADH consumption pathway, the next fermentations were performed in shake flasks. The performance of the HGS7 strain in shake flasks was similar to that in static fermentations, although the glycerol level was slightly reduced and the acetoin level slightly higher (Fig. 3B). Expression of the alternative NADH consumption pathway in strain HGS7, resulting in strain HGS21, pathway, caused a strong reduction in glucose consumption (Fig. 3C). This resulted in lower production of 2,3-BDO and glycerol, without favorably changing the ratio between the two products. To boost the activity of the alternative NADH consumption pathway and reduce glycerol production more strongly, we next replaced the *GPD2* ORF with the *NDE1* ORF, which resulted in strain HGS31. This modification improved glucose consumption and lowered glycerol production to about half of the 2,3-BDO production (Fig. 3D). We next replaced the *GPD1* ORF with the *NDE1* ORF, which resulted in strain HGS38. This completely eliminated glycerol production but again slowed down glucose consumption strongly, although 2,3-BDO production was somewhat higher than in the previous HGS31 strain (Fig. 3E). The 2,3-BDO yield in the HGS21 and HGS38 strains was about 0.33 and about 0.36 g /g glucose, respectively (Fig. 2).

### Downregulation of mitochondrial pyruvate consumption in the alternative oxidase expressing strain

**Fig. 4:**
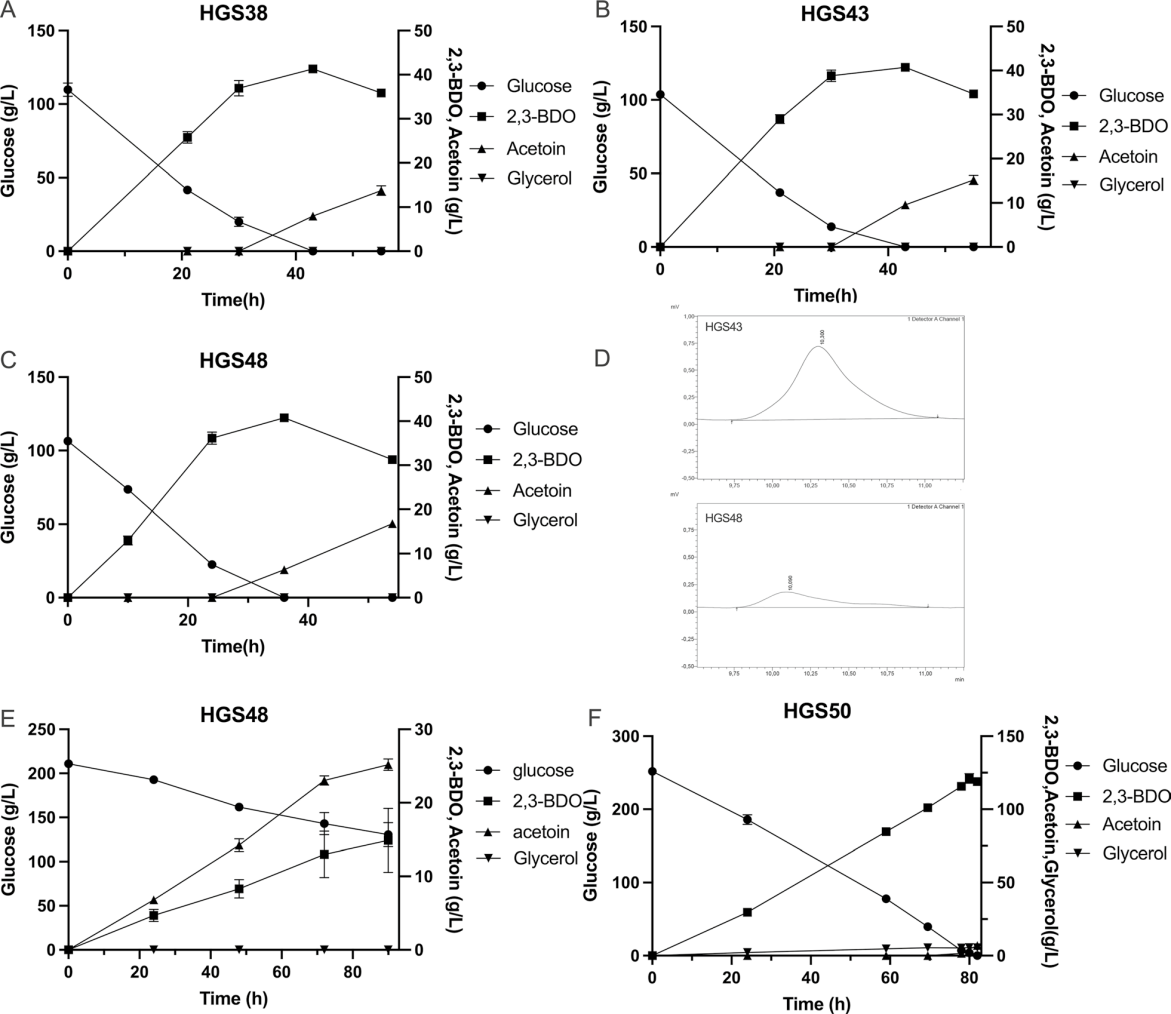
Deletion of the *MPC1* gene, encoding a mitochondrial pyruvate carrier subunit. **A** Production of 2,3-BDO from glucose, with supplementation of 0.5% ethanol, by strain HGS38 in which both *GPD1* and *GPD2* are replaced by *NDE1*. **B** Production of 2,3-BDO from glucose, with supplementation of 0.5% ethanol, by strain HGS43 in which *MPC1* is deleted. **C** Production of 2,3-BDO from 100 g/L glucose in strain HGS48, which has a deletion of *ORA1*. **D** Deletion of *ORA1* eliminated 2,3-dimethylglycerate production. **E** High initial glucose (210 g/L) compromises glucose utilization and 2,3-BDO production in strain HGS48. **F** Low expression of *GPD1* restores complete utilization of a high initial glucose level (250 g/L) and supports production of a high 2,3-BDO titer in strain HGS50

To improve the 2,3-BDO yield in the HGS38 strain expressing the alternative NADH oxidase pathway, we stimulated the flux of pyruvate into the 2,3-BDO synthesis pathway by deleting the *MPC1* gene, encoding a subunit of the mitochondrial pyruvate carrier, resulting in strain HGS43. Since this caused incomplete glucose consumption, although it increased the 2,3-BDO yield from about 0.36 to about 0.50 g/g glucose (Fig. 2), we added 0.5% ethanol in the medium to overcome a possible C2-deficiency. This resulted in complete utilization of the glucose and a significantly higher yield compared to the HGS38 strain in the same glucose + ethanol medium (Fig. 4A, B). In the presence of 0.5% ethanol, it was about 0.45 and 0.48 g/g glucose in the HGS38 and HGS43 strains, respectively (Fig. 2).

### Elimination of 2,3-dimethylglycerate production and osmosensitivity in the alternative oxidase expressing strain

We next deleted the *ORA1* gene, of which the product reduces (S)-α-acetolactate to 2,3-dimethylglycerate and thus competes with AlsD-mediated (R)-acetoin production [47]. This strongly reduced with 80% the 2,3-dimethylglycerate production of the resulting strain HGS48 in medium with 100 g/L glucose (Fig. 4C, D), while the 2,3-BDO yield was not significantly increased (Fig. 2B). The performance of strain HGS48 was also evaluated with an initial glucose level of 210 g/L with the aim of enhancing the final 2,3-BDO titer. However, this resulted in a dramatic reduction of glucose consumption and 2,3-BDO production as well as in a huge increase in acetoin production (Fig. 4E). We supposed that this was due to osmosensitivity of the strain caused by the elimination of glycerol production [48]. Hence, we re-introduced expression of *GPD1* but from the weak *CYC1* promoter [49] and integration in the Mk20 integration site, resulting in strain HGS50. This restored complete consumption of the 250 g/L initial glucose, while maintaining glycerol production at a low level of 11.10 g/L and acetoin at an insignificantly low level (Fig. 4F). Under these conditions, the HGS50 strain produced a 2,3-BDO titer of 121.04 g/L with a productivity of 1.57 g/L.h (0.08 g/L.h per gCDW) and a yield of 0.48 g/g glucose (Fig. 2). When complete utilization of the ethanol for 2,3-BDO production is included, the yield drops somewhat to 0.475 g/g glucose + ethanol.

### Downregulation of glycerol production using the cytosolic NoxE enzyme for consumption of excess NADH

**Fig. 5:**
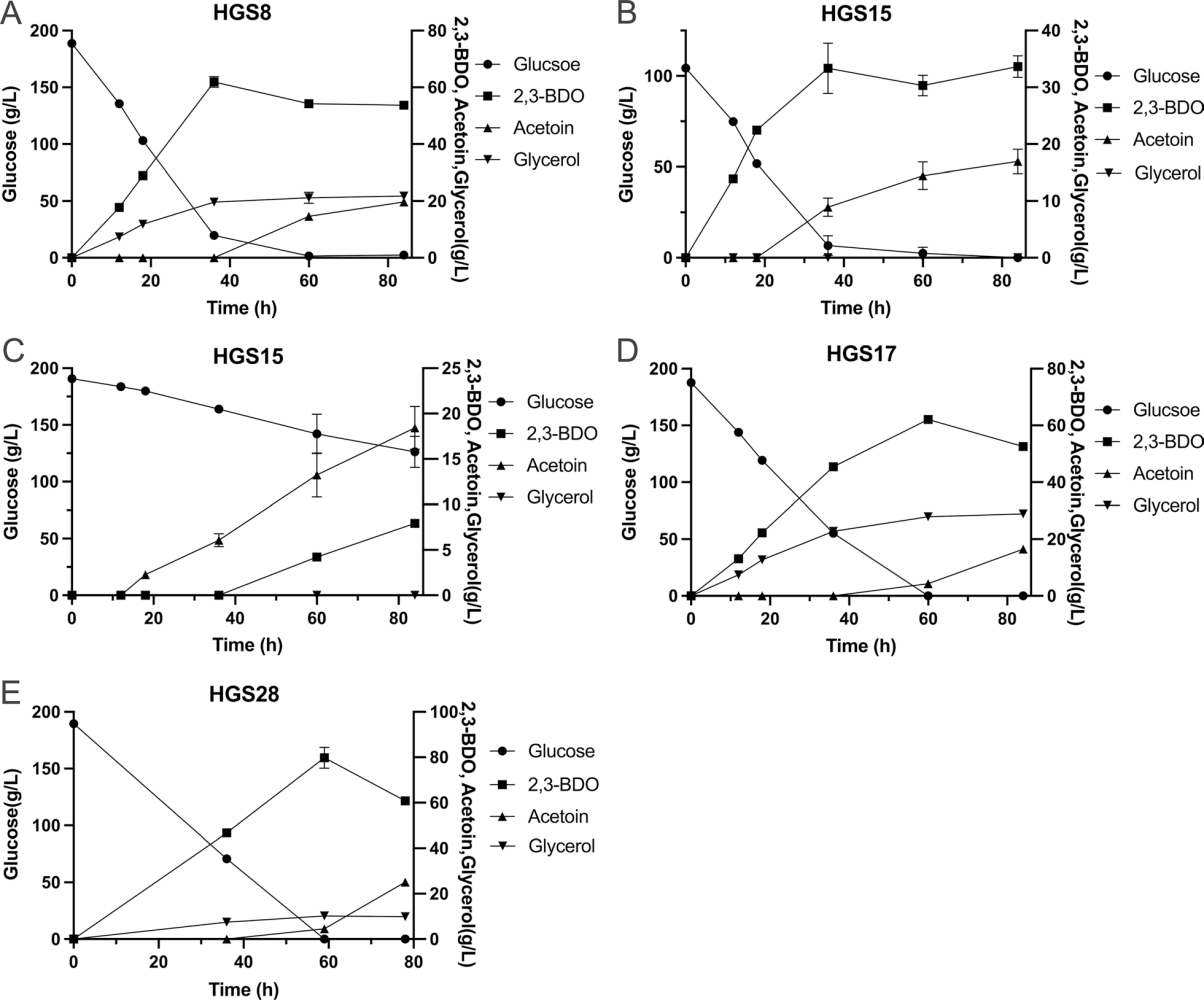
Expression of the cytosolic *NoxE* enzyme for consumption of excess NADH and downregulation of glycerol production. **A** Production of 2,3-BDO from glucose in the HGS8 strain expressing the *NoxE* gene from *Lactococcus lactis* encoding a water-forming NADH oxidase. **B** Production of 2,3-BDO from 10% glucose in the HGS15 strain with deletion of *GPD1* and *GPD2*. **C** Production of 2,3-BDO from 19% glucose in the HGS15 strain with deletion of *GPD1* and *GPD2*. **D** Production of 2,3-BDO from 19% glucose in the HGS17 strain with replacement of the *GPD1* with the *CYC1* promoter. **E** Production of 2,3-BDO from 19% glucose in the HGS28 strain with expression of *GPD1* from the *CYC1* promoter in the Mk20 site

In the second of the two parallel approaches, we expressed the previously utilized *NoxE* gene from *Lactococcus lactis*, encoding a water-forming NADH oxidase, in the HGS7 strain, resulting in strain HGS8. The latter strain showed strong reduction of glycerol production from 69.80 g/L in the HGS7 strain to about 21.76 g/L (Fig. 5A). Next, we deleted the *GPD1* and *GPD2* genes, resulting in strain HGS15, in which glycerol production was completely eliminated. The initial glucose level of 100 g/L was completely consumed in less than 40 h, after which acetoin started to accumulate (Fig. 5B). 2,3-BDO production by strain HGS15 was also evaluated with an initial glucose level of 190 g/L, which resulted in very slow and incomplete glucose utilization (Fig. 5C). This again was likely due to osmosensitivity because of the complete absence of glycerol synthesis. Hence, we next replaced in the HGS8 strain the *GPD1* promoter by the weak *CYC1* promoter and deleted *GPD2*, resulting in strain HGS17. This restored rapid glucose utilization from the initial level of 190 g/L, which was complete in 60 h. At that time the strain had accumulated 63.25 g/L 2,3-BDO but also still a high level of 26.86 g/L glycerol and afterwards started to accumulate acetoin at the expense of 2,3-BDO (Fig. 5D). We also constructed a similar strain but with the *CYC1p-GPD1* construct inserted in another genomic position in strain HGS15, at the Mk20 site, instead of the original *GPD1* site. The resulting strain, HGS28, also consumed all glucose within 60 h but accumulated 79.80 g/L 2,3-BDO and only 9.89 g/L glycerol at the time point of 60 h, after which the strain also started to accumulate acetoin at the expense of 2,3-BDO (Fig. 5E).

### Elimination of 2,3-dimethylglycerate production in the strain expressing the cytosolic NoxE enzyme for consumption of excess NADH

**Fig. 6:**
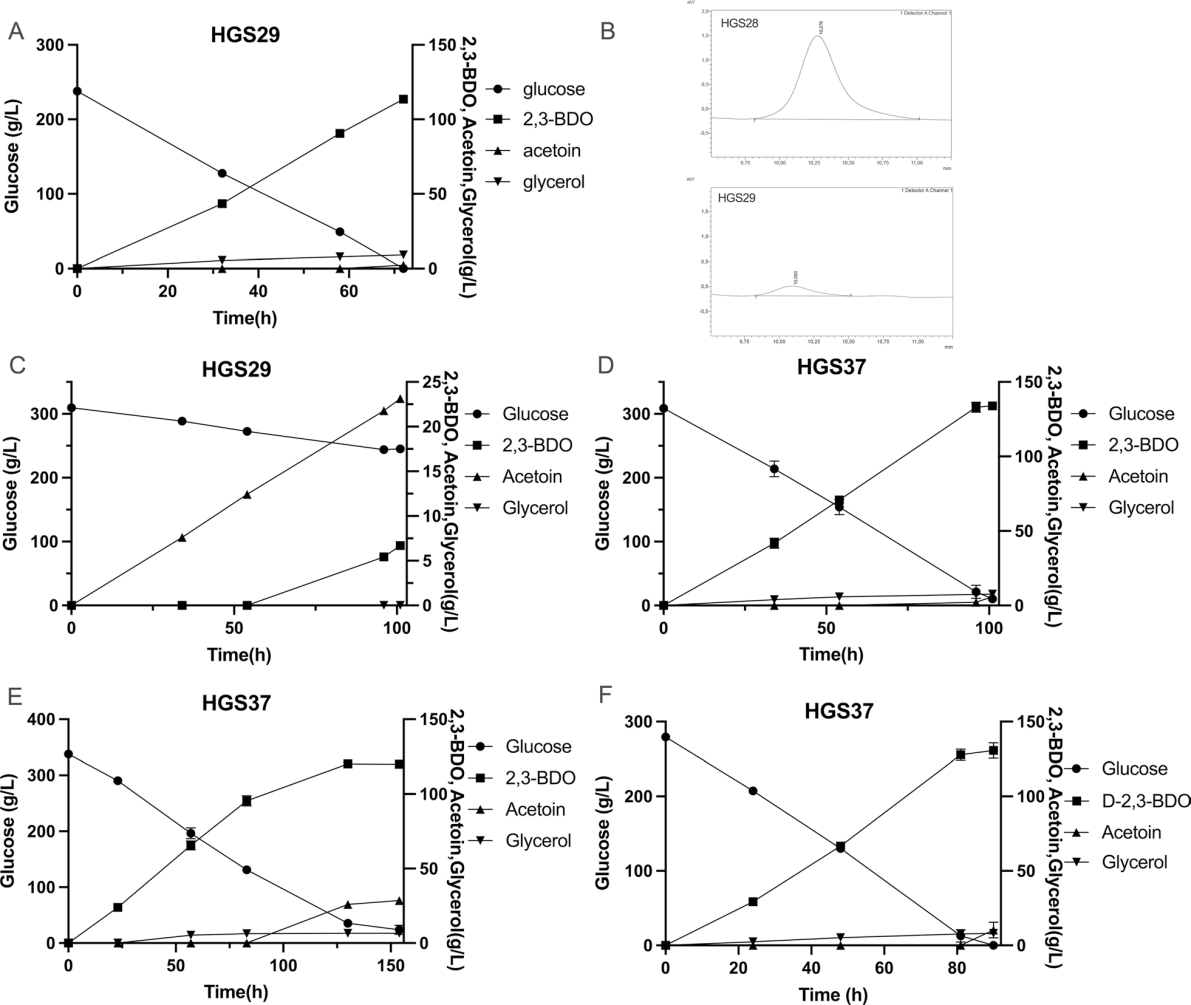
Elimination of 2,3-dimethylglycerate production in the strain expressing the cytosolic *NoxE* enzyme and restoration of high glucose tolerance by increasing glycerol uptake. **A** Production of 2,3-BDO from 23% glucose in the strain HGS29 with deletion of *ORA1.* **B** Deletion of *ORA1* eliminated 2,3-dimethylglycerate production. **C** Production of 2,3-BDO from 31% glucose in the strain HGS29 with deletion of *ORA1*. **D** Production of 2,3-BDO from 31% glucose in the strain HGS37 expressing the CaStl1 glycerol symporter. **E** Production of 2,3-BDO from 34% glucose in the strain HGS37 expressing the CaStl1 glycerol symporter. **F** Production of 2,3-BDO from 28% glucose in the strain HGS37 expressing the *CSTL1* glycerol symporter

We next deleted the *ORA1* gene in strain HGS28 to eliminate 2,3-dimethylglycerate production (Fig. 6A), resulting in strain HGS29. We evaluated that strain at higher initial glucose levels with the aim of reaching higher final 2,3-BDO titers. An initial glucose level of 240 g/L was completely consumed in 70 h and resulted in a final 2,3-BDO concentration of 113.55 g/L with just 2.25 g/L acetoin and 9.03 g/L glycerol produced (Fig. 6A). On the other hand, when an initial glucose level of 310 g/L was used, glucose utilization was severely compromised and 2,3-BDO production reached only 23 g/L (Fig. 6C).

### Further enhancement of high glucose tolerance in the strain expressing the cytosolic NoxE enzyme for consumption of excess NADH

To enable utilization of higher initial glucose levels in order to reach higher final 2,3-BDO titers, we expressed the *STL1* gene from *Candida albicans* (named as *CSTL1*) [50], encoding a glycerol/H^+^ symporter, CaStl1, by insertion in the Mk119 site in the HGS29 strain, resulting in strain HGS37. That strain consumed an initial glucose level of 310 g/L in 100 h, accumulating 133.91 g/L 2,3-BDO, with minimal levels of acetoin (5.88 g/L) and glycerol (7.75 g/L) being produced (Fig. 6D). Utilization of a higher initial glucose level of 340 g/L started to slow down glucose consumption, 23.68 g/L glucose was left after 154 h and only 119.99 g/L 2,3-BDO being produced (Fig. 6E). This was apparently due to the fact that accumulation of acetoin started before all glucose was consumed. The production of 28.56 g/L acetoin after 154 h compromised higher 2,3-BDO accumulation. Glycerol production was still very low with only 6.51 g/L (Fig. 6E). The highest productivity of 1.58 g/L.h (0.11 g/L.h per gCDW) was obtained with an initial glucose level of 28%, reaching a 2,3-BDO titer of 130.64 g/L and a yield of 0.48 g/g glucose (Fig. 6F).

## Discussion

The production of 2,3-BDO from sugar substrates by microbial cell factories poses several challenges. The first major challenge is the redox imbalance in the pathway. The conversion of a glucose molecule into a 2,3-BDO molecule by microbial fermentation produces one NADH molecule in excess. Without any additional adjustment, this causes a huge production of glycerol, which reaches even higher levels than that of the 2,3-BDO produced (Fig. 1B, C). The expression of the gene *NoxE* from *Lactococcus lactis*, encoding a water-forming NADH oxidase, has been used for the first time in 2015 to address the redox imbalance in 2,3-BDO production [37]. Since the conversion of acetoin to 2,3-BDO also uses NADH (Fig. 1A), care should be taken that the expression of *NoxE* does not compromise 2,3-BDO yield by increasing acetoin levels.

We have used 2 alternative approaches to address the redox imbalance: expression of *NoxE* encoding NADH oxidase as previously performed [35, 37, 38, 47, 51], and a new approach consisting of the combined overexpression of *NDE1*, encoding mitochondrial external NADH dehydrogenase [42], and *AOX1*, encoding a heterologous alternative oxidase [43, 52, 53]. The *NoxE* expression in HGS37 resulted in a similar 2,3-BDO productivity (1.58 g/L.h) compared to expression in HGS50 of the combination pathway with the alternative oxidase (1.57 g/L.h), but not when expressed per gCDW: 0.11 g/L.h per gCDW for the former versus 0.08 g/L.h per gCDW for the latter. HGS37 could use YPD medium without addition of ethanol to overcome the C2 deficiency in the pdc-minus strain. On the other hand, the supplementation of ethanol to the YPD for HGS50 facilitated the growth and resulted in higher biomass. Hence, the C2-deficiency may be a factor in causing the difference in productivity when expressed per gDCW. In both cases, overexpression of the NADH consuming enzymes should not be too high in order to avoid the onset of acetoin accumulation. The expression of two more copies of *NoxE* did not result in a difference in 2,3-BDO production. Also the combination of *NoxE* and expression of the Nde1/Aox1 alternative oxidase pathway did not enhance 2,3-BDO production. Probably, the activity of the 2,3-BDO synthesis pathway itself may be limiting. For oxygen consumption, the following equations were calculated. 1. for HGS37: 1 mol glucose + 0.47 mol O_2_ = 0.94 mol 2,3-BDO + 0.06 mol glycerol + 1.88 mol CO_2_, and 2. for HGS50: 1 mol glucose + 0.48 mol O_2_ = 0.96 mol 2,3-BDO + 0.04 mol glycerol + 1.92 mol CO_2_ [21, 48, 54].

Glycerol production could be eliminated completely by deletion of both *GPD1* and *GPD2* (Fig. 3A). However, this makes the yeast highly osmosensitive and therefore compromises the accumulation of a high 2,3-BDO titer from a high initial glucose level. The use of 10% glucose already caused a problem with only part of the glucose being consumed. We addressed the osmosensitivity problem in two ways. We engineered low expression of *GPD1* using the weak *CYC1* promoter and a different insertion site in the genome, which resulted in low glycerol production and greatly restored osmotolerance. In addition, we expressed the heterologous glycerol/H^+^ symporter, Stl1 from *Candida albicans*, in a strain with low glycerol production, which successfully supported re-uptake of glycerol leaked into the medium. This resulted in a further improved osmotolerance allowing complete consumption of 31% glucose and production of 134 g/L 2,3-BDO in 100 h (Fig. 6C). With an initial level of 34% glucose, however, glucose utilization was slower (150 h) and slightly less complete, resulting in a final 2,3-BDO titer of 120 g/L. The reduction of the 2,3-BDO production was also due to onset of acetoin production before all glucose was consumed. Possibly, the higher osmostress with 34% glucose forced more carbon flux into glycerol production, reducing the amount of NADH available for conversion of acetoin to 2,3-BDO. Possibly, higher expression of CaSTL1 may increase the re-uptake of glycerol leaked into the medium further, improving osmotolerance and 2,3-BDO productivity and titer at even higher glucose levels.

Conversion of glucose into 2,3-BDO does not suffer from significant by-product formation. The elimination of 2,3-dimethylglycerate synthesis could increase the 2,3-BDO yield since 2,3-BDO and 2,3-dimythlglycerate share the same precursor, α-acetolactate [47]. The small amounts of 2,3-dimethylglycerate formed could indeed be eliminated by deletion of the *ORA1* gene. Although this strongly reduced with 80% the 2,3-dimethylglycerate production (Figs. 4C, 6A), it only significantly increased the 2,3-BDO yield in strain HGS29 (0.48 g/g glucose), compared to its parent HGS28 (0.44 g/g glucose), while in strain HGS48 the increase was only 0.01 g/L compared to its parent HGS43 and not significant.

Since uptake of pyruvate into the mitochondria competes with the conversion of pyruvate to α-acetolactate, we decided to reduce mitochondrial pyruvate uptake. The mitochondrial pyruvate carrier (MPC) mediates pyruvate uptake and comprises Mpc1 and Mpc2 during fermentative growth, or Mpc1 and Mpc3 during respiratory growth [55–57]. Deletion of the *MPC1* gene reduces the utilization of pyruvate by the mitochondria and thus can increase the 2,3-BDO titer [58]. Normally, only a low level of pyruvate is consumed by mitochondria during fermentative growth because of glucose repression. However, in this study, the deletion of *MPC1* increased the yield of 2,3-BDO from 0.36 g/g glucose in strain HGS38 to 0.50 g/g glucose in strain HGS43 in YPD medium. This indicates that in our strain background the pyruvate consumed by the mitochondria was a large part of the total pyruvate produced. Likely, the expression of the alternative oxidase enhances the pyruvate uptake and consumption by the mitochondria because the alternative oxidase causes leakage of electrons for oxidation of ubiquinol directly with oxygen, which reduces feedback-inhibition by electron chain transport activity on the TCA cycle activity. Although deletion of Mpc1 effectively enhanced the yield of 2,3-BDO, ethanol had to be added in the medium, likely because the problems caused by the C2-deficiency were strengthened (Figs. 2, 4A, B). The effect was lower in the NoxE expressing strain, likely because NoxE bypasses respiration, while it was more pronounced in the strain with the Nde1/Aox1 alternative oxidase pathway because mitochondrial pyruvate increases the flux in the electron transport chain, which competes with the flux through the Nde1/Aox1 alternative oxidase pathway (Fig. 3A).

The consecutive genetic modifications that we introduced in the two parallel strain lineages resulted in a gradual increase in 2,3-BDO yield (Fig. 2). The HGS50 strain showed the best performance of all the strains we constructed. It produced 2,3-BDO with a yield of 0.48 g/g glucose, which is 96% and thus very close to the maximum theoretical yield. It is the highest 2,3-BDO yield ever reported in batch and fed-batch fermentation with yeast cell factories (Table 3). The titers of 121 g/L with HGS50 and 134 g/L with HGS37 are the highest ever reported for batch fermentations of 2,3-BDO (Table 3). When HGS50 or HGS37 would be used in fed-batch fermentation they may exceed the previously reported highest titer of 178 g/L [31]. This assumption is based on previous papers in which a high titer obtained with batch fermentations in erlenmeyer culture could be further increased with fed-batch fermentations in bioreactors [31, 38, 59]. Some previously reported yeast strains display higher productivity, but they have active *GPD* genes, which results in higher glycerol levels compromising yield (Table 3) [31, 38]. On the other hand, further improvement in productivity of the HGS50 and HGS37 strains might be possible. Higher expression of CStl1 in HGS37 may give the strain better tolerance to high glucose levels (e.g. 34%). A weaker promoter compared to that of *CYC1*, together with higher *CSTL1* expression, could further decrease net glycerol production. In spite of this, the performance of HGS50 and HGS37 is probably high enough already for successful industrial implementation in commercial 2,3-BDO production. With bacterial cell factories, higher titers have been obtained but the use of bacteria in large-scale non-sterile industrial production is cumbersome amongst others because of the lack of robustness and difficulty of addressing contamination. On the other hand, it has to be mentioned also that the downstream processing of 2,3-BDO is expensive because of its high boiling point and high hydrophilicity. It can easily entail more than half of the production cost [60].

**Table 3:** Titer, yield, productivity, residual glycerol, fermentation mode and initial glucose level for 2,3-BDO production by different engineered *S. cerevisiae* cell factory microorganisms reported in the literature and in this study

Reference	2,3-BDO titer (g/L)	Yield (g/g glucose)	Productivity (g.L^−1^.h^−1^)	By-product (Glycerol) (g/L)	Batch or Fed batch	Initial glucose (g/L)
[33]	2.29	0.113	–	–	Batch	–
[32]	96.2	0.28	0.39	± 30	Fed batch	100
[37]	32.0	0.367	0.403	6.00	One batch	90
[35]	72.9	0.41	1.43	0.34	Fed Batch	95
[38]	154.3	0.404	1.98	34.00	Fed batch	330
[59]	132.4	0.34	0.41	25.3	Fed batch	120
[61]	81.0	0.27	0.16	71.8	Fed batch	100.0
[31]	178.0	0.335	1.88	> 90	Fed batch	100.0
HGS37 (this study)	130.64	0.48	1.58	8.06	Batch	280
HGS37 (this study)	133.91	0.45	1.33	7.75	Batch	310
HGS37 (this study)	119.99	0.36	0.99	6.51	Batch	340
HGS50 (this study)	121.04	0.48	1.57	10.99	Batch	250

## Conclusions

We have been able to successfully engineer an industrial yeast strain for 2,3-BDO production displaying a 96% yield with glucose as substrate, close to the maximum theoretical yield, a very high titer of 120-130 g/L in batch fermentation, which likely can be further enhanced in fed-batch fermentation, and a high productivity of 1.58 g/L.h. This performance for the three crucial specifications combined is probably adequate for successful implementation of the strain in commercial 2,3-BDO production by yeast fermentation.

## Data Availability

All data have been stored on dedicated computers at KU Leuven. All data and yeast strains are available upon request.

## Abbreviations

2,3-BDO: 2,3-Butanediol
MEK: Methyl ethyl ketone
YPD: Yeast extract peptone glucose
CDW: Cell dry weight
